# Imatinib associated discoid lupus erythematosus in a patient with chronic myeloid leukemia

**DOI:** 10.1016/j.jdcr.2024.08.020

**Published:** 2024-09-01

**Authors:** Priya Sarlashkar, Christian Carr, Meghan Heberton

**Affiliations:** Department of Dermatology, University of Texas Southwestern Medical Center, Dallas, Texas

**Keywords:** cutaneous toxicities, discoid lupus erythematosus, drug reaction, imatinib, oncodermatology

## Introduction

Chronic myeloid leukemia (CML) is a hematopoietic stem cell disorder characterized by the presence of the Philadelphia chromosome, a reciprocal translocation between chromosomes 9 and 22 resulting in the BCR-ABL1 fusion gene. This genetic abnormality drives uncontrolled cell proliferation and is a hallmark of CML. Imatinib, a tyrosine kinase inhibitor, specifically targets the BCR-ABL1 oncoprotein.^1^ Previously reported adverse events to imatinib include superficial edema, maculopapular rash, pigmentary changes, lichenoid eruptions, and photosensitivity.^2^

Here, we present a case of a patient with CML who developed discoid lupus erythematosus (DLE) associated with imatinib.

## Case report

A 39-year-old female with CML was referred to the oncodermatology clinic due to periorbital edema and a facial rash. The rash appeared soon after she began taking imatinib for CML. The patient also endorsed new onset photosensitivity after imatinib initiation. She started with 400 mg every other day for the first 2 months and then increased to 400 mg daily. The increase in dosage led to worsening rash and photosensitivity noted by the patient. The patient had also started lisinopril and metoprolol during this time frame but noticed the rash and photosensitivity prior to the start of these medications and specifically after imatinib. She had no history of autoimmune or other dermatologic diseases.

**Fig 1:**
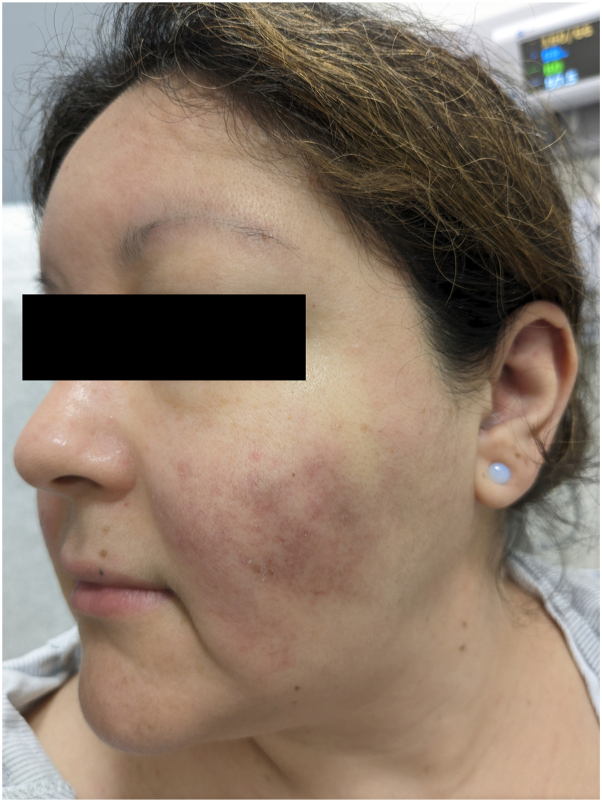
Hyperpigmented, scaly, violaceous patches on bilateral cheeks sparing the nasolabial folds.

**Fig 2:**
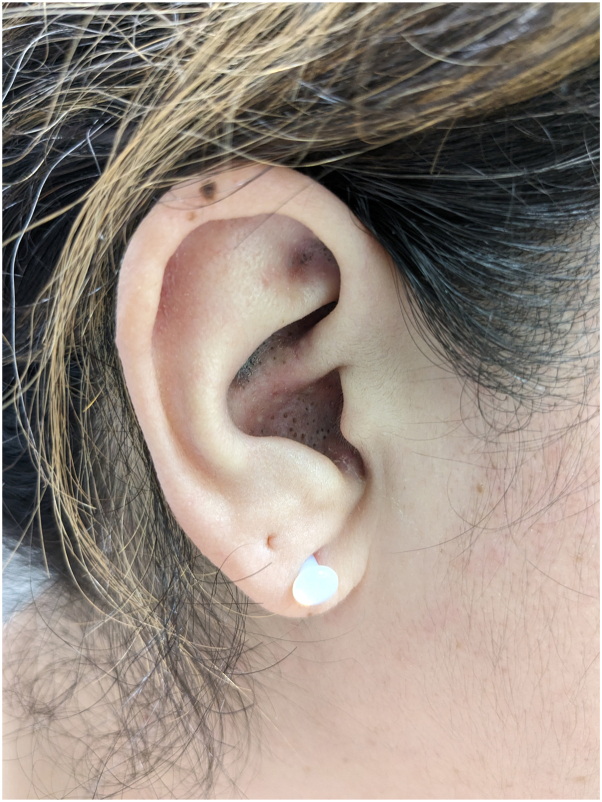
Hyperpigmented, *pink* patches with follicular plugging in conchal bowls and scaphoid fossa.

The patient used over-the-counter hydrocortisone cream for this rash without improvement. Physical examination revealed hyperpigmented and violaceous scaly patches on her bilateral cheeks in a malar distribution with sparing of the nasolabial folds (Fig 1). Hyperpigmented plaques with follicular plugging were also noted in the conchal bowls and scaphoid fossae (Fig 2). A clinical diagnosis of DLE was favored. A deep shave biopsy from the left scaphoid fossa confirmed hyperkeratosis, follicular plugging and dilatation, thickening of the basement membrane, and focal vacuolar degeneration of the basal layer consistent with DLE. Laboratory tests revealed a positive antinuclear antibody titer (1:320) with a speckled staining pattern and weakly positive SS-A. SS-B, RNP, SCL-70, anti-Smith, antihistone, and ds-DNA were negative. C3 and C4 were within normal limits.

A urinalysis, complete blood count, and comprehensive metabolic panel were reassuring, and she had no arthritis. A thorough review of systems for systemic lupus erythematosus (SLE) was negative and the patient did not meet criteria per European League Against Rheumatism/American College of Rheumatology criteria.^3^ A diagnosis of DLE affecting the face and ears was made. Imatinib was favored as a provoking agent given the temporal association and dose dependency of her symptoms and findings. The patient was prescribed clobetasol 0.05% cream for her ears, hydrocortisone 2.5% cream for her face, and strict photoprotection was recommended. Given persistent rash and symptoms on 2 month follow up, hydroxychloroquine 200 mg twice daily was added. The patient remained on imatinib to treat her CML.

## Discussion

This case is a novel presentation of imatinib associated DLE. Our patient developed clinical signs and symptoms of DLE with temporal and dose-dependent association with imatinib. DLE presents with well-defined, erythematous, scaly plaques that can lead to scarring and dyspigmentation. Drug-induced DLE is rare and has been reported in association with anti-tumor necrosis factor α drugs, antihypertensives, and chemotherapeutic agents, but not previously with imatinib.^4^

The mechanism by which DLE can manifest following imatinib exposure remains unclear but may involve imatinib’s ability to suppress regulatory T cells (Tregs). Tregs play a crucial role in promoting self-tolerance to prevent autoimmunity. Prior research has suggested that imatinib can deplete Treg cells, disrupting immune tolerance.^5^^,^^6^ While this process improves antitumor immunity, it may unmask underlying autoimmune conditions such as DLE in susceptible individuals. It has been postulated that drug induced autoimmunity is idiosyncratic, and its presentation may be influenced by factors including a patient’s genetic predisposition, interaction with other drugs, and environmental influences.^7^

This case highlights critical implications in terms of diagnosis and management of adverse events associated with cancer therapies. Imatinib is a key component of the therapeutic regimen for CML. Given our patient’s grade 2 presentation, imatinib was continued with appropriate interventions to control her rash. Cessation of imatinib upon diagnosis would have been pre-emptive in this case and limited critical cancer therapy. Diagnosis with dermatology expertise was necessary to correctly triage and work up her presentation.

An important component in the work up of a patient presenting with DLE includes evaluation for concomitant SLE. Although SLE development has not been reported with imatinib exposure, there have been cases of dasatinib-associated SLE.^8^^,^^9^ Dasatinib is another BCR-ABL tyrosine kinase inhibitor that has additional inhibitory effects on SRC family kinases, c-KIT, platelet-derived growth factor receptor, and EphA2.^1^ While not every patient taking imatinib warrants assessment for SLE, those who present with signs of cutaneous lupus should receive appropriate longitudinal monitoring. Prior research has identified age of <25 at time of DLE diagnosis, phototype V to VI, and antinuclear antibody titer ≥1:320 as risk factors for the progression of DLE to SLE.^10^ Timely intervention requires monitoring serological markers, such as anti-dsDNA and anti-Sm, and conducting a thorough review of systems for SLE criteria.

The impact of imatinib-induced DLE on management can be significant. Mild cases of DLE may escalate and lead to irreversible scarring which would necessitate critical interdisciplinary treatment discussions. Dermatologists and oncologists should be aware of this adverse effect to allow for appropriate management while preserving critical cancer therapy.

## Conflicts of interest

Dr Heberton has served on an advisory board for Blueprint Medicines. Authors Sarlashkar and Carr have no conflicts of interest to declare.
